# Community-Wide Genotyping of *Batrachochytrium dendrobatidis* in Ecuadorian Forests

**DOI:** 10.1007/s10393-025-01716-y

**Published:** 2025-05-05

**Authors:** Wesley J. Neely, M. D. M. Moretta-Urdiales, Utpal Smart, Ryan L. Lynch, Juan Manuel Guayasamin, Shawn F. McCracken, David Rodriguez

**Affiliations:** 1 Department of Biology, Texas State University, 601 University Drive, San Marcos, TX USA; 2 Department of Natural Sciences, Texas A&M University – San Antonio, San Antonio, TX USA; 3 Third Millennium Alliance, Fremont, CA USA; 4 Laboratorio de Biología Evolutiva, Instituto de Investigaciones Biológicas y Ambientales BIOSFERA, Universidad San Francisco de Quito, Quito, Ecuador; 5 Department of Life Sciences, College of Science, Texas A&M University – Corpus Christi, Corpus Christi, TX USA

**Keywords:** andes, amphibian biodiversity, chytrid, disease dynamics, genotyping efficiency

## Abstract

The amphibian chytrid fungus *Batrachochytrium dendrobatidis* (*Bd*) is a cosmopolitan pathogen with numerous distinct lineages. The global panzootic lineage (*Bd*-GPL) is the most widespread and virulent lineage and is responsible for many recorded amphibian declines. Mapping the extent of *Bd*-GPL and other more established lineages is important for predicting disease dynamics in amphibian communities. Ecuador is the most biodiverse country per unit area for amphibian taxa and, thus, a priority for studies on genotypic diversity of *Bd*. In this study, we tested skin swab samples collected from 464 individual amphibians across coastal, Andean montane, and Amazonian forests, for the presence of two *Bd* lineages known to be present in South America: the globally-distributed *Bd*-GPL and the Brazilian-endemic *Bd*-Asia2/Brazil lineage. By using a discriminatory qPCR SNP assay, we found widespread prevalence of *Bd*-GPL in Ecuador in diverse host taxa. Genotyping efficiency was 36% in this study, meaning that one in every three swabs that tested positive for *Bd* in infection assays were successfully genotyped. Through this study, we provide further support for the presence of a single *Bd* lineage in this neotropical biodiversity hotspot.

**Supplementary Information:**

The online version contains supplementary material available at https://doi.org/10.1007/s10393-025-01716-y.

## Introduction

Globally, emerging infectious diseases (EIDs) in humans, livestock, and wildlife have increased in prevalence (Jones et al., 2008; Nova et al., 2022). While the etiological agents causing infections can include bacteria, viruses, parasites, or prions, fungi are responsible for several of the most prominent examples in vertebrates (Fisher et al., 2016). These include white-nose syndrome in bats (Blehert et al., 2009), snake fungal disease (Lorch et al., 2016), and amphibian chytridiomycosis attributed to species of *Batrachochytrium* (Berger et al., 2016). These aquatic, zoosporic fungi infect the skin of amphibians, leading to hyper- or hypokeratosis in susceptible taxa, which can disrupt osmoregulation (Van Rooij et al., 2015; Voyles et al., 2009). Investigations of host–pathogen dynamics of the amphibian chytrid fungus, *Batrachochytrium dendrobatidis* (*Bd*), have demonstrated epizootic patterns of introduction and spread leading to host declines, followed by enzootic maintenance of pathogens in asymptomatic or sub-clinical carriers post-invasion (Briggs et al., 2010; Carvalho et al., 2017; LaBumbard et al., 2020). One challenging factor in the study of *Bd* is the presence of multiple genetically distinct lineages, which can be difficult to diagnose morphologically in microscopic fungi (Schloegel et al., 2012). Yet, the development and application of genetic tools, such as DNA sequencing and discriminatory single nucleotide polymorphism (SNP) genotyping, has allowed a better understanding of regional and global pathogen diversity, especially in the amphibian–chytrid system (Byrne et al., 2019; Farrer et al., 2011; Ghosh et al., 2021; Jenkinson et al., 2016; O’Hanlon et al., 2018).

Intra-specific pathogen diversity is an important factor to consider when studying disease dynamics because it can modulate the effects of diseases on hosts (Bruns et al., 2012; Greenspan et al., 2018; Jenkinson et al., 2018; Taylor et al., 1997). For example, different genotypes of the crown rust fungus (*Puccinia coronata*) produce differing numbers of infective spores, enabling greater infectivity (Bruns et al., 2012). In a clinical setting, diverse genotypes of infective yeast (*Candida glabrata*) have displayed differences in drug susceptibility (Badrane et al., 2023). Similarly, diverse *Bd* genotypes display wide variation in virulence (Greenspan et al., 2018; Jenkinson et al., 2018; Muletz-Wolz et al., 2019). The global panzootic lineage (*Bd*-GPL), for example, is considered to be more virulent than regional enzootic lineages, like *Bd*-Asia2/Brazil (Becker et al., 2017; O’Hanlon et al., 2018; Ribeiro et al., 2019; Rosenblum et al., 2012). However, hybridization between these two lineages has been recorded (Schloegel et al., 2012), with hybrids showing higher virulence than either parent lineage (Greenspan et al., 2018).

Studies using standard qPCR assays based on the ITS1-5.8S region (Boyle et al., 2004) have shown that *Bd* is prevalent in neotropical anuran communities (Becker et al., 2016; Carvalho et al., 2017; Guayasamin et al., 2014; Rebollar et al., 2014). This method provides presence and infection intensity data, but it does not help discriminate between described lineages, which include *Bd*-GPL, *Bd*-Asia1, *Bd*-Asia2/Brazil, *Bd*-CH, and *Bd*-CAPE (Farrer et al., 2011; O’Hanlon et al., 2018; Rosenblum et al., 2013). Past genotyping efforts in South American countries have been relatively limited in scope considering the high diversity of amphibian hosts on the continent (Byrne et al., 2019; Carvalho et al., 2023; Jenkinson et al., 2016; Rodriguez et al., 2014). Contemporary and retrospective studies indicate that only *Bd*-GPL and *Bd*-Asia2/Brazil have been detected in South America, with *Bd*-Asia2/Brazil and a hybrid isolate (*Bd*-GPL x *Bd*-Asia2/Brazil) only occurring in parts of Brazil (Jenkinson et al., 2016; Rodriguez et al., 2014). *Bd*-GPL has been reported more broadly across some South American countries (Burrowes et al., 2020; Byrne et al., 2019; James et al., 2015; Smart et al., 2024), but the spatial resolution of genotype data remains low. Expanded *Bd* genotyping in this region of the world is needed to pinpoint areas of concern, particularly those with heightened risk of hybridization where multiple genotypes might potentially co-occur (Greenspan et al., 2018; Schloegel et al., 2012).

Infection dynamics can also vary between *Bd* genotypes based on the identity of the infected host (Byrne et al., 2022). Consequently, studies focusing on genotypic diversity of *Bd* in one host species might underrepresent the true range of genotypic diversity present within an ecosystem. Sharp differences in *Bd* transcription profiles between host species have also been identified (Ellison et al., 2017), which may indicate a significant role of host diversity in driving *Bd* lineage evolution. Therefore, studies aiming to classify genotypic diversity would benefit from incorporating community-wide sampling for more rigorous pathogen surveillance. Neotropical regions with high amphibian species richness (e.g., Ecuador) should be of highest priority for studies on host-genotype associations.

Ecuador is an ecologically diverse country and hosts the third highest amphibian richness by area globally, with more than 600 species (AmphibiaWeb, 2024). While amphibians in this area are primarily threatened by habitat loss (Ortega-Andrade et al., 2021), *Bd* infection is widespread and may also threaten susceptible species (Guayasamin et al., 2014; McCracken et al., 2009; Narváez-Narváez et al., 2021; Urgiles et al., 2021; Vega-Yánez et al., 2024). Previous studies have identified *Bd*-GPL as the only lineage present in Ecuador (Byrne et al., 2019; Smart et al., 2024); however, these studies were restricted in terms of number of host species and geographic extent. Specifically, in a global study of *Bd* genotypic diversity, Byrne et al. (2019) genotyped seven museum-preserved anurans from six sites across Ecuador. Later, Smart et al. (2024) conducted a more extensive survey of an amphibian community, but only at a single site in the Ecuadorian Amazon. Therefore, geographically broad community-wide genotypic surveys in this hyper-diverse area would be valuable in clarifying *Bd* genotype–host associations and performing surveillance for *Bd*-Asia2/Brazil outside of Brazil.

In this study, we genotyped *Bd* infections in forest-associated amphibians from three different Ecuadorian ecoregions (i.e., coastal, Andean montane, and Amazonian forest) to identify the *Bd* lineage(s) present in these diverse communities. We leveraged skin swab samples collected at six different sites across three years to classify *Bd* genotypic diversity among host species and location. We also aimed to identify the efficiency of our method in genotyping *Bd* from positive skin swab extracts. With these data, we attempted to identify locations where multiple genotypes potentially co-occur or provide additional evidence for the presence of a single *Bd* strain in this highly biodiverse region. Given that this region has previously experienced *Bd* invasion and a subsequent epizootic wave (Lips et al., 2008), comprehensive analyses of the diversity of this pathogen can provide insights into past infection patterns.

## Methods

**Figure 1:**
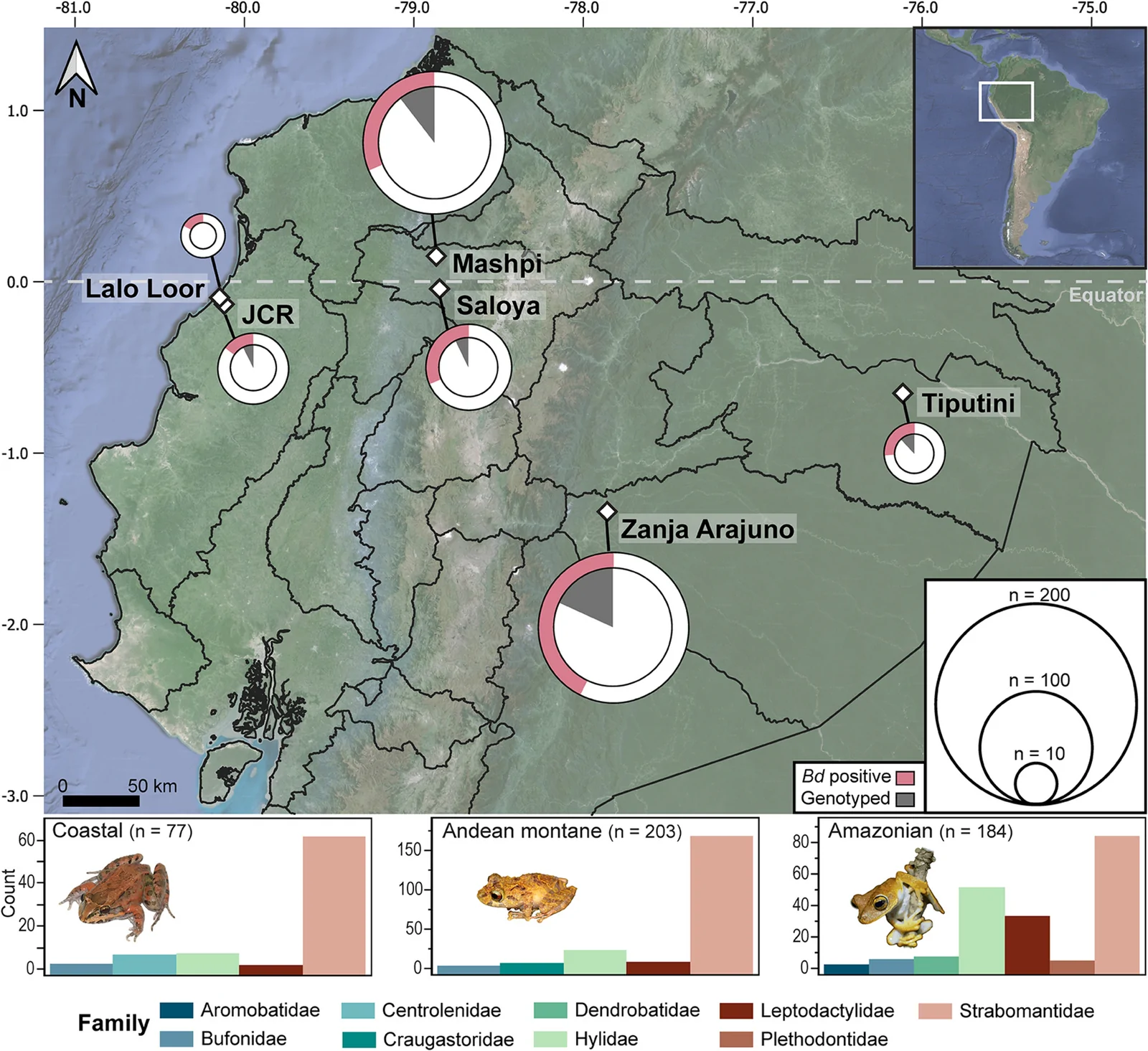
Map of sampling locations for *Batrachochytrium dendrobatidis* (*Bd*) infections across Ecuador. Names correspond to the following sample sites: Lalo Loor = Reserva Bosque Seco Lalo Loor, JCR = Jama-Coaque Reserve, Mashpi = Mashpi Lodge, Saloya = Privately owned property, Zanja Arajuno = Zanja Arajuno Ecological Center, and Tiputini = Tiputini Biodiversity Station. Pie charts show *Bd* prevalence (outer circle with red shading) and number genotyped (inner circle with gray shading). Sizes of pie charts reflect sample sizes. Bars show the relative diversity of frog families sampled in each Natural Region: Coastal (JCR and Lalo Loor), Andean montane (Mashpi and Saloya), and Amazonian (Zanja Arajuno and Tiputini). Frogs pictured are *Leptodactylus* sp. (left), *Pristimantis* sp. (middle), and *Boana* sp. (right).

We sampled amphibian communities at six protected or privately owned sites across Ecuador from June 2021 through December 2023 (Fig. 1; Supplementary Fig. 1). Primary habitat at all sites consisted of humid tropical rainforest (Instituto Geografico Militar 2010). Lalo Loor Dry Forest Reserve (Lalo Loor) and Jama-Coaque Reserve (JCR) are in low-elevation coastal forest (< 700 m.a.s.l.) and characterized by distinct dry and rainy seasons, with abundant deciduous and semi-deciduous flora. Mashpi Lodge (Mashpi) and Saloya are in western Andean montane humid forest and characterized by high humidity and precipitation throughout the year. Zanja Arajuno Ecological Center (Zanja Arajuno) is in middle-elevation Amazonian forest and characterized by high annual rainfall and relatively stable temperatures throughout the year. Tiputini Biodiversity Station (Tiputini) is in low-elevation Amazonian forest (< 300 m.a.s.l.) and characterized by low seasonal variation, high temperatures, and high humidity. At each site, we conducted visual and acoustic encounter surveys and captured any detected amphibians in clean plastic bags. To test for *Bd* infections, we swabbed amphibians using sterile rayon swabs (MW113, Medical Wire) following standardized protocols consisting of 10 passes down the venter and five passes on the plantar surface of each foot (Hyatt et al., 2007). We dry-stored swabs in sterile 1.5-mL tubes at 4 °C until DNA extraction.

We extracted DNA from swabs using 50 µL of PrepMan™ Ultra Sample Preparation Reagent (Applied Biosystems) following previously described procedures (Becker et al., 2016). We then diluted DNA 1:10 with nuclease-free water before quantitative polymerase chain reaction (qPCR). We conducted qPCRs to amplify *Bd* DNA (Boyle et al., 2004), with the following recipe per sample: 6.25 µL TaqMan Fast Advanced Master Mix (ThermoFisher), 0.50 µL bovine serum albumin at 400 ng/µL, 1.375 µL nuclease-free water, 0.625 µL ITS1-3 Chytr forward primer (18 µM), 0.625 µL 5.8S Chytr reverse primer (18 µM), 0.625 µL Chytr MGB2 probe (5 µM), and 2.5 µL of diluted template DNA. We included standards ranging from 0.5 to 5,000 zoospore equivalents and no template controls (NTC) to check for contamination of reagents. We ran qPCRs using 48-well portable magnetic induction real-time PCR cyclers (Mic; Bio Molecular Systems) and the following protocol: 95 °C for 5 min, then 50 cycles of 95 °C for 5 s, 60 °C for 10 s, and 72 °C for 10 s (Acquire on Green).

For *Bd* genotyping qPCR assays, we used the following recipe per sample: 5.0 µM TaqMan Fast Advanced Master Mix (ThermoFisher), 0.5 µM 20X Bdmt_26360 SNP Assay (Jenkinson et al., 2018), 1.0 µM nuclease-free water, and 2.5 µL of diluted template DNA. We ran genotyping qPCRs on the Mic instruments using the following cycling protocol: 95 °C for 5 min, then 50 cycles of 95 °C for 5 s, 60 °C for 20 s (Acquire on Green and Yellow), and 72 °C for 10 s. To characterize the association between *Bd* load and genotyping success, all samples were included in the genotyping assay, regardless of the infection assay results.

We calculated genotyping efficiency by dividing the percent of successfully genotyped samples by the percent of *Bd*-positive samples. To assess the relationship between genotyping success, categorized as either not genotyped (0) or genotyped (1), and *Bd* infection status, we ran two generalized linear models with binomial distributions using the glmmTMB package in R version 4.2.2 (Brooks et al., 2024; R Core Team 2022). The first model included *Bd* infection status as the predictor and was run using all extracted samples (*n* = 464). The second model included log10-transformed *Bd* loads as the predictor for *Bd*-positive samples only (*n* = 147). To compare genotype assay fluorescence to *Bd* infection loads (log10-transformed), we fit a linear regression for the samples that were successfully genotyped (*n* = 51). We visualized results using the ggplot2 package in R version 4.2.2 (R Core Team 2022; Wickham, 2016). The R code used to run our statistical analyses, calculate prevalences with binomial confidence intervals, and visualize results is included as a supplementary file.

## Results

**Figure 2:**
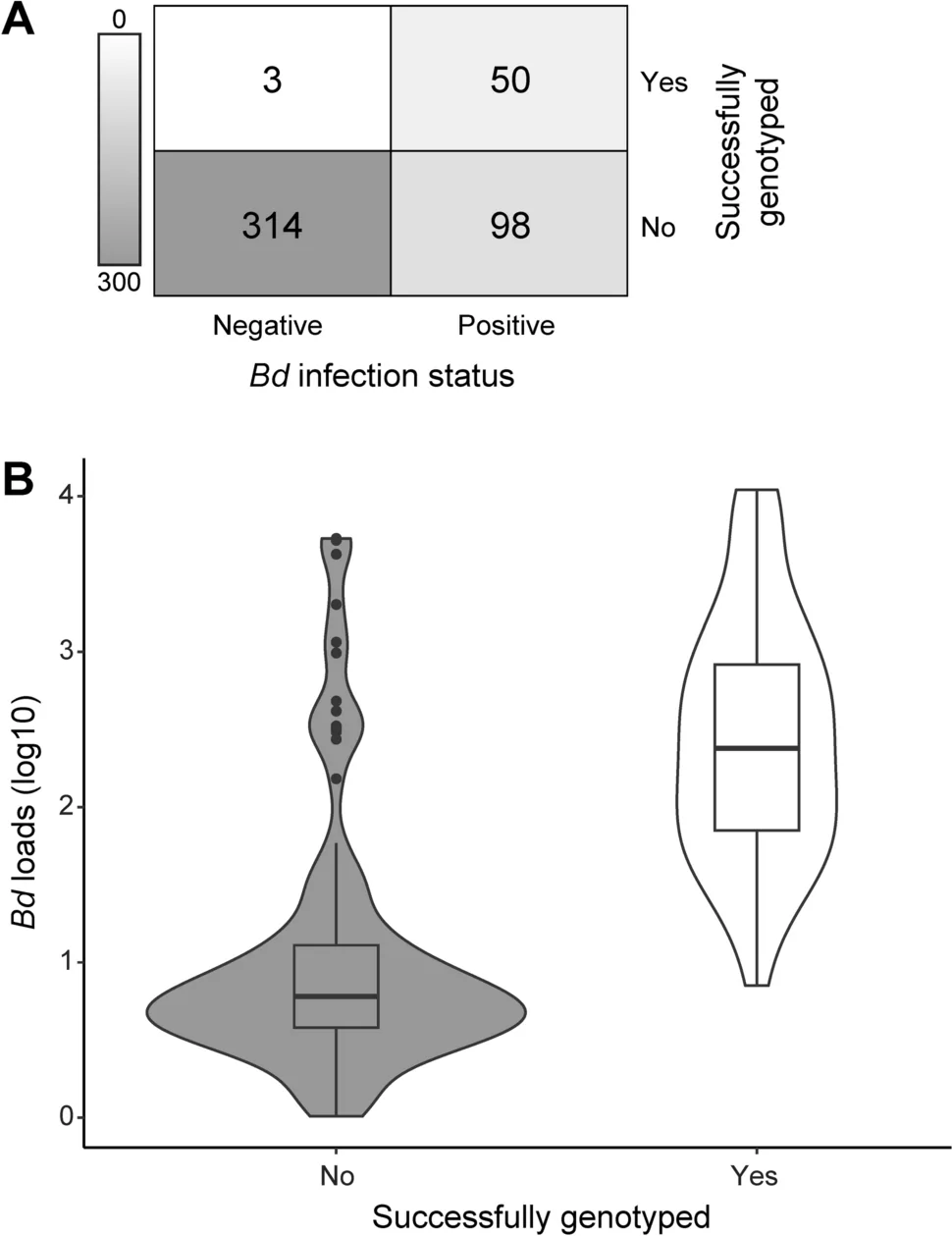
Visualizations showing the relationship between *Batrachochytrium dendrobatidis* (*Bd*) infection and genotyping success. **A** Heatmap showing the relationship between *Bd* infection status and genotyping success. Darker shades of gray indicate more matching observations. **B** Violin plot with inlaid box plots showing the distribution of *Bd* loads between genotyped (*Bd*-GPL) and non-genotyped amphibian skin swabs from Ecuador. *Bd* loads were log10-transformed to correct for heavy right skew characteristic of pathogen load data.

In total, we analyzed skin swabs from 464 amphibians comprising 22 genera and 9 families (Table 1). We were unable to identify all individuals to species, particularly those in the genus *Pristimantis*. While we morphologically identified 46 species across the 22 genera, this number underrepresents the full diversity in our dataset and thus for accuracy all analyses are at the genus and family levels. *Bd* prevalence was 32% (95% CI = 28–36%) across all sites and taxa, and 11% (95% CI = 9–15%) of all samples were successfully genotyped (Fig. 1). Genotyping efficiency was 36% overall (Table 1). Excluding taxa with fewer than five positive detections, efficiency was highest for the genera *Engystomops* (53%), *Boana* (50%), and *Pristimantis* (35%; Supplementary Table 1). *Bd* prevalence and genotyping success were both highest at Zanja Arajuno private reserve (*Bd*: 43.0%, 95% CI = 34.7–51.5%; Genotype: 17.6%, 95% CI = 11.7–24.9%; Fig. 1; Table 1), but genotyping efficiency was highest at JCR (50%; Fig. 1; Table 1). Genotyping success was significantly associated with *Bd* infection status (Estimate = 3.70 ± 0.53, *P* < 0.001; Fig. 2A) and *Bd* loads (Estimate = 1.65 ± 0.26, *P* < 0.001; Fig. 2B). We identified 53 positive detections as *Bd*-GPL, and three genotyped samples tested negative for *Bd*. Genotype assay fluorescence was positively associated with *Bd* infection loads for successfully genotyped samples (*β* = 0.57 ± 0.10, *R*^2^ = 0.40, *P* < 0.0001; Supplementary Fig. 2).

**Table 1:** *Batrachochytrium dendrobatidis* (*Bd*) Prevalence and Genotyping Across the Six Sampling Locations in Ecuador.

Location	Latitude	Longitude	Elevation (m)	Generic richness	*N*	Positive	Genotyped	Average loads (95% CI)	% Infected (95% CI)	% Genotyped (95% CI)	Efficiency (%)
JCR	− 0.1168	− 80.1225	280	8	54	8	4	93.0 (0–853.6)	14.8 (6.6–27.1)	7.4 (2.1–17.9)	50
Lalo Loor	− 0.0778	− 80.1501	35	4	23	4	0	232.0 (0–1,996)	17.4 (5–38.8)	0 (0–14.8)	0
Mashpi	0.1656	− 78.8792	920	5	134	42	14	38.0 (0–407.6)	31.3 (23.6–39.9)	10.4 (5.8–16.9)	33
Saloya	− 0.0804	− 78.8292	1,135	6	69	21	5	267.7 (0–2,194.5)	30.4 (19.9–42.7)	7.2 (2.4–16.1)	24
Tiputini	− 0.6384	− 76.1434	240	11	42	11	5	19.2 (0–148.2)	26.2 (13.9–42)	11.9 (4–25.6)	45
Zanja Arajuno	− 1.3319	− 77.8771	960	13	142	61	25	331.5 (0–3,414.7)	43.0 (34.7–51.5)	17.6 (11.7–24.9)	41
**Overall**					**464**	**147**	**53**	**176.6 (0–2,120)**	**31.7 (27.5–36.1)**	**11.4 (8.7–14.7)**	**36**

Columns show coordinates, elevation (meters), the number of amphibian genera sampled (Generic richness), the total number of individuals sampled (N), the number of *Bd*-positive individuals (Positive), the number successfully genotyped (Genotyped), average *Bd* infection loads (in zoospore equivalents) with 95% confidence intervals, *Bd* prevalence (% Infected) with 95% binomial confidence intervals, genotyping success (% Genotyped) with 95% binomial confidence intervals, and genotyping efficiency (Efficiency). Location names correspond to the following sample sites: Lalo Loor = Reserva Bosque Seco Lalo Loor, JCR = Jama-Coaque Reserve, Mashpi = Mashpi Lodge, Saloya = Privately owned property, Zanja Arajuno = Zanja Arajuno Ecological Center, and Tiputini = Tiputini Biodiversity Station.

## Discussion

We found widespread prevalence of the global panzootic lineage of *Bd* (*Bd*-GPL) across Ecuadorian amphibian communities, which is consistent with findings from the two previous studies in this country (Byrne et al., 2019; Smart et al., 2024). *Bd*-GPL is widely distributed, documented from amphibians worldwide (Byrne et al., 2019; Farrer et al., 2011), and it has the potential to outcompete more regional strains due to its high virulence and possible tolerance of broader environmental conditions (Becker et al., 2017; Belasen et al., 2022; Byrne et al., 2022; Farrer et al., 2011; James et al., 2015; Schloegel et al., 2012). Our results affirm the widespread prevalence of *Bd*-GPL in Ecuador and suggest that this genotype might have been associated with historical declines in this region (Lips et al., 2008; Scheele et al., 2019), but targeted museum sampling around the time of declines is needed to support this hypothesis. As EIDs like *Bd* continue to threaten biodiversity worldwide (Jones et al., 2008), our findings contribute valuable nationwide data on the genotypic diversity of this pathogen in Ecuador (Bruns et al., 2012; Greenspan et al., 2018).

In this study, we used a mitochondrial SNP assay, and we were only able to differentiate *Bd*-GPL from *Bd*-Asia2/Brazil (Jenkinson et al., 2018), so it is possible that some of our samples identified as *Bd*-GPL could represent a hybrid lineage with *Bd*-GPL as the maternal parent (Carvalho et al., 2023; Ghosh et al., 2021). However, this is less likely given we did not detect *Bd*-Asia2/Brazil, which has been shown to be restricted to southeastern Brazil in South America (Byrne et al., 2019; Schloegel et al., 2012). If the latter strain is present in Ecuador, it is likely in very low proportions, and additional surveillance efforts could help detect future introduction events. Subsequent research should prioritize community-wide surveys at more localities and genomic loci, to improve our understanding of the spatial distribution of *Bd* lineages.

Genotyping efficiency was lower in this study (36% overall) than another study (60%) in the Ecuadorian Amazon sampled during the same season (Smart et al., 2024). When comparing only samples from the same locality (Tiputini Biodiversity Station), genotyping efficiency was still lower in our study (45%), which could be due to the source material and DNA extraction methods employed. Smart et al. (2024) used DNA from archived (ca. 2008) toe clips extracted using the DNeasy Blood & Tissue Kit (Qiagen, Inc.), while we used skin swabs collected following standardized methodology (Hyatt et al., 2007) and extracted using PrepMan Ultra™ (Applied Biosystems). This suggests that toe clip samples may perform better than skin swabs for genotyping *Bd*-infected amphibians. Smart et al. (2024) also had higher *Bd* prevalence (58%) and average loads (16,057 zoospore equivalents) than found at the same site in this study (Prevalence = 26%, Loads = 19 z.e.; Table 1), further supporting higher performance of toe clips for *Bd* detection. However, due to variation when generating standard curves, caution is warranted when comparing *Bd* loads. For example, one study found that toe clips were more sensitive than swabs for *Bd* detection via qPCR (Voordouw et al., 2010). However, other studies comparing both methods for *Bd* detection found no differences (Burrowes et al., 2011; Hyatt et al., 2007), yet similar tests specifically for genotyping are still needed. Alternatively, extractions with PrepMan Ultra™ may underrepresent *Bd* infections, particularly for low-load infections as previously reported (Bletz et al., 2015).

We found variable genotyping efficiency of our assay among host taxa. We had low sample sizes for many genera, likely biasing our estimates of genotyping efficiency for those groups. Among taxa with enough positives to allow comparison, *Boana* and *Engystomops* had the highest genotyping efficiency, with half of positive frogs successfully genotyped. This means that future genotyping efforts could target these genera for greater success. However, this approach might underrepresent the range of genotypic diversity present if host-specific infection is prevalent (Byrne et al., 2022); thus, target approaches should be used to compliment community-wide surveys. Lack of genotyping success for some species could indicate cryptic diversity of *Bd* that failed to match either genotype in our mitochondrial assay, although, no close relatives to *Bd* were detected in a targeted metagenomic survey of the mycobiome of amphibians from Ecuador (Jervis et al., 2020). Targeting taxa displaying high infection loads (e.g., *Trachycephalus*) could be fruitful for detecting new invasions of other lineages, hybrid *Bd* lineages, or the more recently discovered chytrid fungus *B. salamandrivorans* by DNA sequencing or additional assays (Carvalho et al., 2023; Jenkinson et al., 2018). Host identity is, therefore, another important factor to consider when surveying *Bd* genotypic diversity.

Three samples that tested negative for *Bd* infection were successfully genotyped. This result could be due to false negatives in the *Bd* infection qPCR assay or false positives in the *Bd* genotyping qPCR assay. Alternatively, this may indicate the presence of cryptic genotypes that are genetically divergent from both *Bd*-GPL and *Bd*-Asia2/Brazil. The *Bd* genotyping assay targets the mitochondrial genome, while the *Bd* detection assay targets ribosomal genes. The copy number of ribosomal genes is known to be variable for *Bd* (Longo et al., 2013); thus, it is possible that low-load infections were present and the mitochondrial genome enabled detection in the genotyping assay, but low ribosomal ITS copy number may have inhibited detection in the infection assay. By comparing genotype fluorescence to *Bd* loads; however, we found that both assays have a strong positive correlation, with some variability likely driven by these differences between the two assays.

## Conclusions

Our *Bd* genotyping survey across multiple forest types in Ecuador sheds light on taxa-specific genotyping efficiency and underscores the importance of community-wide genotyping efforts. Further investigations sequencing *Bd* at more loci would be useful to accurately assess the diversity of this pathogen in the region. Given these findings, it is imperative to prioritize targeted genotyping efforts and proactive conservation plans to safeguard Ecuador's amphibian biodiversity from the threat of rapidly evolving pathogens, which may have undergone multiple introductions in the past (Rodriguez et al., 2014).

## Supplementary Information

Below is the link to the electronic supplementary material.

Supplementary file1 (DOCX 81 KB)
