# Supplementary material for: Community-Wide Genotyping of Batrachochytrium dendrobatidis in Ecuadorian Forests
Source: Ecohealth. 2025 May 5;23(3):488–97. doi: 10.1007/s10393-025-01716-y (PMC13582374; doi:10.1007/s10393-025-01716-y)
Supplement: Supplementary file 1 [file 10393_2025_1716_MOESM1_ESM.docx]

**Supplemental Materials**

**Supplementary table 1**: *Batrachochytrium dendrobatidis* (*Bd*) prevalence and genotyping for amphibian families and genera. For families with only one representative genus, the data is combined into a single entry. Columns show the sample sites (Sites), total number of individuals sampled (N), the number of *Bd*-positive individuals (Positive), the number successfully genotyped (Genotyped), Average *Bd* infection loads (in genome equivalents) with 95% confidence intervals, *Bd* prevalence (% Infected) with 95% binomial confidence intervals, genotyping success (% Genotyped) with 95% binomial confidence intervals, and genotyping efficiency (Efficiency). Sample site codes are as follows: All = observed at all six sites, JCR = Jama-Coaque Reserve, LL = Reserva Bosque Seco Lalo Loor, M = Mashpi Lodge, S = Saloya privately-owned property, T = Tiputini Biodiversity Station, and ZA = Zanja Arajuno Ecological Center.

| **Family** | **Genus** | **Sites** | **N** | **Positive** | **Genotyped** | **Average Loads**  **(95% CI)** | **% Infected**  **(95% CI)** | **% Genotyped**  **(95% CI)** | **Efficiency** |
| --- | --- | --- | --- | --- | --- | --- | --- | --- | --- |
| Aromobatidae | *Allobates* | T,ZA | 2 | 1 | 0 | 4.6 (0 – 17.8) | 50.0 (2.6 – 97.4) | 0 (0 – 65.8) | 0% |
| Bufonidae | *Rhinella* | All | 9 | 0 | 0 | 0 (0 – 0) | 0 (0 – 29.9) | 0 (0 – 29.9) | - |
| Centrolenidae |  |  | 6 | 0 | 0 | 0 (0 – 0) | 0 (0 – 39.0) | 0 (0 – 39.0) | 0% |
|  | *Espadarana* | JCR | 3 | 0 | 0 | 0 (0 – 0) | 0 (0 – 56.1) | 0 (0 – 56.1) | - |
|  | *Hyalinobatrachium* | JCR | 3 | 0 | 0 | 0 (0 – 0) | 0 (0 – 56.1) | 0 (0 – 56.1) | - |
| Craugastoridae | *Craugastor* | M | 5 | 1 | 0 | 1.2 (0 – 6.6) | 20.0 (1.0 – 62.4) | 0 (0 – 43.4) | 0% |
| Dendrobatidae | *Ameerega* | T | 7 | 4 | 1 | 8.5 (0 – 31.3) | 57.1 (25.0 – 84.2) | 14.3 (0.7 – 51.3) | 25% |
| Hylidae |  |  | 80 | 19 | 8 | 59 (0 – 517) | 23.8 (15.8 – 34.1) | 10.0 (5.2 – 18.5) | 42% |
|  | *Agalychnis* | ZA | 1 | 0 | 0 | 0 (0 – 0) | 0 (0 – 94.9) | 0 (0 – 94.9) | - |
|  | *Boana* | M,S,T,ZA | 35 | 8 | 4 | 44.3 (0 – 345.1) | 22.9 (12.1 – 39.0) | 11.4 (4.5 – 26.0) | 50% |
|  | *Dendropsophus* | S,ZA | 7 | 5 | 1 | 153.7 (0 – 886.3) | 71.4 (35.9 – 91.8) | 14.3 (0.7 – 51.3) | 20% |
|  | *Hyloscirtus* | M | 8 | 2 | 1 | 193.5 (0 – 1284.1) | 25.0 (7.1 – 59.1) | 12.5 (0.6 – 47.1) | 50% |
|  | *Osteocephalus* | T | 6 | 0 | 0 | 0 (0 – 0) | 0 (0 – 39.0) | 0 (0 – 39.0) | - |
|  | *Phyllomedusa* | ZA | 13 | 1 | 0 | 0.9 (0 – 7.5) | 7.7 (0.4 – 33.3) | 0 (0 – 22.8) | 0% |
|  | *Scinax* | JCR,S,ZA | 5 | 2 | 2 | 10.3 (0 – 52.5) | 40.0 (11.8 – 76.9) | 40.0 (11.8 – 76.9) | 100% |
|  | *Smilisca* | JCR | 3 | 0 | 0 | 0 (0 – 0) | 0 (0 – 56.1) | 0 (0 – 56.1) | - |
|  | *Trachycephalus* | LL | 2 | 1 | 0 | 240.2 (0 – 919.6) | 0.5 (2.6 – 97.4) | 0 (0 – 65.8) | 0% |
| Leptodactylidae |  |  | 40 | 19 | 9 | 45.7 (0 – 277.3) | 47.5 (32.9 – 62.5) | 22.5 (12.3 – 37.5) | 47% |
|  | *Adenomera* | T,ZA | 3 | 1 | 1 | 12.1 (0 – 53.9) | 33.3 (1.7 – 79.2) | 33.3 (1.7 – 79.2) | 100% |
|  | *Engystomops* | T,ZA | 27 | 15 | 8 | 66.2 (0 – 339.8) | 55.6 (37.3 – 72.4) | 29.6 (15.9 – 48.5) | 53% |
|  | *Leptodactylus* | JCR,S,T,ZA | 10 | 3 | 0 | 0.5 (0 – 2.5) | 30.0 (10.8 – 60.3) | 0 (0 – 27.8) | 0% |
| Strabomantidae |  |  | 312 | 103 | 36 | 242 (0 – 2592) | 33.0 (27.8 – 38.5) | 11.5 (8.2 – 15.6) | 35% |
|  | *Barycholos* | JCR,LL | 3 | 0 | 0 | 0 (0 – 0) | 0 (0 – 56.1) | 0 (0 – 56.1) | - |
|  | *Oreobates* | T,ZA | 4 | 2 | 0 | 1.8 (0 – 6.2) | 50.0 (15.0 – 85.0) | 0 (0 – 49.0) | 0% |
|  | *Pristimantis* | All | 304 | 101 | 35 | 247.6 (0 – 2623.2) | 33.1 (27.8 – 38.7) | 11.8 (8.4 – 16.0) | 36% |
| Plethodontidae | *Bolitoglossa* | T,ZA | 4 | 0 | 0 | 0 (0 – 0) | 0 (0 – 49.0) | 0 (0 – 49.0) | - |

**Supplementary figure 1**: Sampling timeline across Ecuador at six protected or privately-owned sites from June 2021 through December 2023.

**Supplementary figure 2**: Scatterplot showing *Batrachochytrium dendrobatidis* (*Bd*) loads in relation to genotype assay fluorescence for samples that were genotyped (Red) or not genotyped (Grey) for amphibians sampled in coastal, Andean montane, and Amazonian forests of Ecuador. SNP assay Bdmt_26360 from Jenkinson et al. (2018).
